# Study on the changes in TSH, TPO-Ab and other indicators due to Vitamin D deficiency in Pregnant Women with subclinical hypothyroidism in the first trimester

**DOI:** 10.12669/pjms.36.6.1982

**Published:** 2020

**Authors:** Xue Zhou, Ben Li, Chao Wang, Zhihong Li

**Affiliations:** 1 Xue Zhou, Department of Endocrinology, Baoding first Central Hospital, Baoding, Hebei 071000, P.R.China; 2 Ben Li, Dept. of Thoracic Surgery, Affiliated Hospital of Hebei University, Baoding, Hebei 071000, P.R.China; 3 Chao Wang, Department of Endocrinology, Baoding first Central Hospital, Baoding, Hebei 071000, P.R.China; 4 Zhihong Li, Department of Endocrinology, Baoding first Central Hospital, Baoding, Hebei 071000, P.R.China

**Keywords:** Vitamin D, Subclinical hypothyroidism, The first trimester, Thyroid stimulating hormone, High-sensitivity C-reactive protein, Interleukin-1

## Abstract

**Objective:**

To investigate the effect of vitamin D deficiency on the levels of thyroid stimulating hormone (TSH), thyroid peroxidase antibody (TPO-Ab), interleukin-1 (IL-1) and high-sensitivity C-reactive protein (hs-CRP) in pregnant women with early pregnancy complicated by subclinical hypothyroidism.

**Methods:**

A total of 172 pregnant women with subclinical hypothyroidism in the first trimester diagnosed and treated in a given hospital from August 2014 to May 2018 were selected, and their levels of vitamin D were determined. Depending on the abnormality of their vitamin D levels, the study participants were divided into two groups: the study group (vitamin D≤20 ng/L) and the control group (vitamin D>20 ng/L). The levels of TSH, TPO-Ab, IL-1 and hsCRP in the two groups were measured.

**Results:**

The levels of TSH, hsCRP and TPO-Ab in the study group were significantly higher than those in the control group (P<0.05). The comparison between the two groups in terms of IL-1 showed no statistically significant difference.

**Conclusion:**

Vitamin D deficiency in the first trimester is associated with in an increased level of TSH in the first trimester, thereby aggravating subclinical hypothyroidism. The mechanism may be associated with the impact of vitamin D deficiency on hs-CRP and other body inflammation indicators, as well as on thyroid autoantibodies and other immune indicators, but has no effect on IL-1 levels.

## INTRODUCTION

The thyroid is an important endocrine organ that controls human metabolism, and the abnormal secretion of hormones by the thyroid can result in hypothyroidism or hyperthyroidism.^1^ In the first trimester of pregnancy, thyroid hormone is especially crucial. Normally, the thyroid volume would increase by approximately 10% during pregnancy, making triiodothyronine (T3) and thyroxine (T4) levels increase by approximately 50%, manifested as the increase in daily iodine demand and the decrease in the level of thyroid stimulating hormone (TSH) in the first trimester.^2^,^3^ Subclinical hypothyroidism occurring in the first trimester can raise the risk of poor outcome in the second and third trimesters, such as placental abruption, preeclampsia, diabetes and anemia.^4^,^5^ In addition, thyroid hormone is vital to the development of the fetal central nervous system, and subclinical hypothyroidism in the first trimester could induce irreversible damage to the fetal cranial nerve.^6^ In recent years, studies have shown that vitamin D can play an important role in the occurrence and development of thyroid diseases in the first trimester by becoming involved in the regulation of IL-1, hsCRP, leptin and other cytokines.^7^,^8^

The objective of the present study was to investigates the effect of vitamin D deficiency on the changes in the levels of TSH, TPO-Ab, IL-1 and hsCRP in pregnant women with subclinical hypothyroidism (hereinafter referred to as SCH) in the first trimester.

## METHODS

A total of 172 pregnant women with subclinical hypothyroidism in the first trimester who were diagnosed and treated in a given hospital from August 2014 to May 2018 were selected as the research subjects. The inclusion criteria for the study were as follows: those who met the diagnostic criteria for subclinical hypothyroidism in the first trimester set forth by the Endocrine Society of the Chinese Medical Association in *Guidelines for the Diagnosis and Management of Thyroid Disease during Pregnancy and Postpartum*; those for whom the gestational age was less than 13 weeks; those with no personal or family history of genetic diseases; those with no reproductive disease history; and those with a singleton pregnancy. The exclusion criteria for the study were as follows: a previous history of thyroid disease or other endocrine diseases; those with hypertension, chronic kidney disease, cerebrovascular disease or diabetes; those with concomitant allergic disease, malignant tumor or acute infection; or those who took contraceptives, estrogens, glucocorticoids or other drugs that may have damaged the thyroid function over the previous month.

### Ethical Approval

The study was approved by the Institutional Ethics Committee of Baoding first Central Hospital, and written informed consent was obtained from all participants.

### Grouping

25 (OH) D3 is the main circulating form of vitamin D in the blood, therefore the level of 25 (OH) D3 was measured. According to vitamin D deficiency, the subjects were divided into two groups: the study group (vitamin D<20 ng/L) and the control group (vitamin D≥20 ng/L). The levels of TSH, TPO-Ab, hsCRP and IL-1 in the two groups were measured.

### Measurement of Indicators

For the measurement of the indicators, i.e., TPO-Ab, IL-1, hsCRP and TSH, approximately 3 ml of venous blood was collected while the patient was in a fasted state. After 30 min, the blood was centrifuged at 3500 rpm at 40^0^C to isolate the upper serum. Using an automatic biochemical analyzer (Roche), the levels of TPO-Ab, IL-1, hsCRP and TSH in the serum were determined.

### Statistical Methods

For the purpose of statistical analysis, SPSS 19.00 was used. The measurement data were expressed as the mean ± SD. The intergroup comparison of the measurement data was performed with a t-test, and P<0.05 was deemed statistically significant.

## RESULTS

### General Conditions

There were no statistically significant differences between the two groups in terms of age, gestational week, parity and gravidity (P>0.05). Table-I.

**Table-I:** Comparison of General Data (Mean ±SD).

Group	Number of Patients (n)	Age (yrs)	Gestational Week (wks)	Parity (Times)	Gravidity (Times)
Study Group	100	27.82±3.14	6.33±2.19	1.89±1.33	3.10±0.78
Control Group	72	27.10±4.49	6.82±1.48	1.92±0.92	3.02±0.67
T		1.238	-1.646	-0.165	0.703
P		0.218	0.102	0.869	0.483

### Comparison between the Two Groups in Terms of the Observed Indicators

In Study Group and Control Group, the hsCRP levels were 7.92±1.48mg/Land 7.14±1.25 mg/L respectively. There was significant difference between the two groups(P<0.05). TPO-Ab levels were 200.22±7.6U/mL and 186.89±7.1U/mL respectively. There was significant difference between the two groups(P<0.05). IL-1 levels were 88.45±4.23pg/ml and 88.91±5.34pg/ml respectively. There was no significant difference between the two groups(P>0.05). TSH levels were 6.94±0.25mIU/L and 6.11±0.31mIU/L respectively. There was significant difference between the two groups(P<0.05). Table-II.

**Table-II:** Comparison between the two groups in terms of the different observed indicators (Mean±SD).

Group	Number of Patients (n)	hsCRP (mg/L)	TPO-Ab (U/mL)	IL-1 (pg/ml)	TSH (mIU/L)
Study Group	100	7.92±1.48	200.22±7.6	88.45±4.23	6.94±0.25
Control Group	72	7.14±1.25	186.89±7.1	88.91±5.34	6.11±0.31
t		3.634	-2.547	-0.630	19.411
P		<0.001	0.012	0.530	<0.001

## DISCUSSION

The results of this study show that the TSH of the study group was higher than that of the control group, with statistically significant differences, implying that vitamin D deficiency affects the level of TSH in vivo and may be involved in the occurrence of subclinical hypothyroidism in the first trimester.

Among the tissues and organs of the body, the thyroid can regulate the synthesis and metabolism of protein, glucose and fat in the body by secreting an appropriate amount of thyroid hormone, thereby affecting the metabolism, growth and development of the human body. In the first trimester, if TSH fails to adapt to the physiological changes of pregnancy, thyroid dysfunction will happen. Thyroid hormone is vital to the development of the fetal central nervous system, and subclinical hypothyroidism in the first trimester will have a certain influence on both pregnant women and their fetuses and increase the incidence of poor pregnancy outcomes.^9^ Vitamin D deficiency is also involved in various pregnancy disorders. Vitamin D deficiency can increase the reabsorption of Na^+^ by the kidney, reduce prostaglandin and other vasodilators, impair vasodilation, increase vascular resistance and elevate blood pressure.^10^ Vitamin D deficiency can also diminish pulmonary surfactant, postpone the maturity of fetal lungs and finally lead to fetal distress and neonatal asphyxia.^11^ Moreover, pregnant women with vitamin D deficiency tend to have excessive fat on their abdominal wall, and their abdominal strength is poor, therefore they often have uterine atony and an increased probability of cesarean delivery.^12^ Vitamin D deficiency can also cause fetuses to be in a hyperglycemia state for a long time. Fat synthesis is hyperactive, while fat decomposition is suppressed, which often gives rise to fetal macrosomia and premature delivery.^13^,^15^

Both prenatal subclinical hypothyroidism and vitamin D deficiency are common diseases in the first trimester. They can produce adverse effects in pregnant women and fetuses. So, do they have a potential correlation? In recent years, vitamin D deficiency has been shown to potentially affect thyroid function status. Vitamin D plays a role by binding with receptors and activating the responsive genes of receptors, and it is able to initiate the autoimmunity of thyroid cells, thereby avoiding the occurrence of autoimmune thyroid diseases.^12^ When vitamin D deficiency occurs in the body, immune cells will not be suppressed and autoimmunity will occur, making the thyroid involved excessively and leading to thyroid dysfunction.^16^

TPO-Ab is an important antibody reflecting the autoimmune thyroiditis of the human body. The findings of Tamer et al. indicated that the incidence of vitamin D deficiency was 92% among patients with Hashimoto’s thyroiditis and 63% among healthy people, implying that vitamin D deficiency was associated with Hashimoto’s thyroiditis and that the level of vitamin D in the serum of patients with Hashimoto’s thyroiditis was lower than that of healthy people.^17^ In the results of the present study, the TPO-Ab titer of the study group was higher than that of the control group, with a statistically significant difference, suggesting that vitamin D deficiency can affect the autoimmune status of the thyroid and contribute to a higher titer of thyroid antibody.

The hs-CRP is a kind of particular protein generated by human liver cells and an indicator reflecting the inflammatory state of the body. Some studies have revealed that hs-CRP can directly induce endothelial cells to generate plasma plasminogen activator inhibitors and increase their activity, thereby regulating the thyroid function of the body.^18^ The results of this study suggest that the level of hs-CRP in the study group with vitamin D deficiency was higher than that of the control group, and the difference was statistically significant, implying that vitamin D deficiency could increase the inflammatory reaction of the body and may be involved in the occurrence of subclinical hypothyroidism.

IL-1 is a kind of cytokine generated by monocytes, endothelial cells, fibroblasts, and other types of cells in response to infection and has a relationship with the immunity of the body. Studies have shown that IL-1 can play an important role in autoimmune diseases, especially in the onset of Hashimoto’s thyroiditis.^19^,^20^ However, the results of this study show that the difference between the study group and control group in the level of IL-1 was not statistically significant, suggesting that the influence of vitamin D deficiency on thyroid function and immune status may have nothing to do with IL-1.

Therefore, vitamin D deficiency in early pregnancy should be identified by monitoring of 25 (OH) D level and supplements should be provided where indicated.

### Limitation of the study

The number of patients in this study is not large enough, therefore some indicators are not highly specific. The relationship between vitamin D and subclinical hypothyroidism in the first trimester and its possible mechanisms are yet to be discussed and verified through in-depth clinical and fundamental studies.

## CONCLUSION

Vitamin D deficiency in the first trimester is associated with an increased level of TSH in the first trimester, thereby aggravating subclinical hypothyroidism. The mechanism may be associated with the impact of vitamin D deficiency on hs-CRP and other body inflammation indicators, as well as on thyroid autoantibodies and other immune indicators, but has no effect on IL-1 levels.

### Authors’ Contributions:

**ZL** **& XZ:** Designed this study and prepared this manuscript and are responsible and accountable for the accuracy of the work;

**BL:** Collected and analyzed clinical data

**CW:** Significantly revised this manuscript.
